# Variable *in Vivo* and *in Vitro* Biological Effects of Cerium Oxide Nanoparticle Formulations

**DOI:** 10.3389/fphar.2019.01599

**Published:** 2020-01-28

**Authors:** Karin L. Heckman, Ana Y. Estevez, William DeCoteau, Stephanie Vangellow, Samantha Ribeiro, Joseph Chiarenzelli, Bonnie Hays-Erlichman, Joseph S. Erlichman

**Affiliations:** ^1^ Department of Biology, St. Lawrence University, Canton, NY, United States; ^2^ Department of Psychology, St. Lawrence University, Canton, NY, United States

**Keywords:** cerium oxide nanoparticles, oxidative stress, antioxidants, experimental autoimmune encephalomyelitis, macrophages

## Abstract

Cerium oxide nanoparticles (CeNPs) exhibit redox capacity *in vitro* with efficacy in *in vivo* disease models of oxidative stress. Here we compare, in parallel, three CeNP formulations with distinct chemical stabilizers and size. *In vitro* assays revealed antioxidant activity from all the CeNPs, but when administered to mice with a reactive oxygen species (ROS) mediated model of multiple sclerosis, only custom-synthesized Cerion NRx (CNRx) citrate-EDTA stabilized CeNPs provided protection against disease. Detectable levels of ceria and reduced ROS levels in the brains of CNRx CeNP-treated mice imply that these CeNPs' unique properties influence tissue distribution and subsequent biological activity, suggesting why differing CeNP formulations yield different *in vivo* effects in various models. Further, the variation in *in vivo* vs *in vitro* results with these CeNP formulations highlights the necessity for *in vivo* studies that confirm whether the inherent catalytic activity of CeNPs is maintained after transport and distribution within intact biological systems.

## Introduction

Cerium oxide nanoparticles (CeNPs) possess potent antioxidant activity attributable to a crystalline, lattice structure with oxygen vacancies for the acquisition, or release of electrons during fluctuation from a Ce^+3^ to a Ce^+4^ state (Reed et al., 2014). In cell-free and *in vitro* cell/tissue models, this catalytic activity mimics the function of endogenous enzymes catalase (Pirmohamed et al., 2010) and superoxide dismutase (SOD) (Korsvik et al., 2007) in order to neutralize a variety of reactive oxygen species (ROS) molecules, including nitric oxide (Dowding et al., 2012), superoxide (Pirmohamed et al., 2010), and peroxynitrite (Dowding et al., 2013). Like other nanomaterials, CeNPs can be produced by a range of synthesis methods, yielding different particle sizes, surface charges, and zeta potentials. In addition, functionalizing particles with stabilizers and coating materials potentially alters a variety of factors including catalytic activity (Lee et al., 2013; Dunnick et al., 2015), aggregation tendencies (Ould-Moussa et al., 2014), corona formation favoring particles with a negative *in vitro* zeta potential (Patil et al., 2007), likelihood of cellular uptake (Patil et al., 2007), and biodistribution pattern, which also varies with administration route and particle size (Yokel et al., 2009; Hardas et al., 2010; Hirst et al., 2013; Yokel et al., 2013).

Though the toxicological effects of accidental and occupational CeNP exposure have been investigated, CeNPs have increasingly been applied to disease models, particularly those involving oxidative stress (Heckman et al., 2013; Bailey et al., 2016; DeCoteau et al., 2016; Kwon et al., 2016; Naz et al., 2017). The administration of CeNPs has recently been shown to be efficacious in models of traumatic brain injury (Bailey et al., 2016), amyotrophic lateral sclerosis (ALS) (DeCoteau et al., 2016), radiation-induced lung damage (Xu et al., 2016), chronic liver (Oró et al., 2016) or kidney injury (Manne et al., 2015a), peritonitis (Manne et al., 2015b), and obesity (Rocca et al., 2015). Though it is tempting to extrapolate the applicability of these results to *all* CeNPs, even within just these few studies, the particles utilized range from 3–80 nm in size, exhibited variable amounts of aggregation, and were delivered at doses ranging from 0.0007 mg/kg (Xu et al., 2016) to 20 mg/kg (DeCoteau et al., 2016) for mice and 0.05 mg/kg (Bailey et al., 2016) to 0.5 mg/kg (Rocca et al., 2015) for rats. Thus, while different formulations of CeNPs have exhibited antioxidant activity, parallel investigation of the catalytic activity and biological efficacy of CeNPs would strengthen our understanding of how unique characteristics of CeNPs influence their function.

We study custom CeNPs (CNRx) with characteristics distinct from other nanoceria formulations. These CeNPs are relatively small at 1.5–3.0 nm and are stabilized with citrate and EDTA. Though nanomaterials typically adsorb a high number of proteins into their corona (Monopoli et al., 2012), only a relatively small number of proteins adhere to the CNRx CeNPs (Heckman et al., 2014): a profile of molecules that would promote receptor mediated uptake (ApoE) and transcytosis (albumin). These CeNPs exhibit catalase and SOD-like activity *in vitro*, enabling the reduction of ROS levels in murine hippocampal brain slices exposed *ex vivo* to ischemic conditions (Estevez et al., 2019). Further, the CeNPs oppose peroxide or ischemia induced shifts in the oxidation-reduction potential of brain tissue (DeCoteau et al., 2016). This antioxidant activity translates to *in vivo* efficacy in oxidative-stress mediated murine models of multiple sclerosis [experimental autoimmune encephalomyelitis (EAE)] (Heckman et al., 2013) and ALS (DeCoteau et al., 2016). Mice induced with EAE treated intravenously with CNRx CeNPs exhibited reduced clinical disease severity and retained motor function similar to mice treated with a currently prescribed drug, Fingolimod. Reduced intracellular levels of ROS detected in the brains of treated animals support an antioxidant mechanism of protection (Heckman et al., 2013).

Despite the efficacy of the CNRx custom CeNPs in the EAE model, treatment of EAE mice with another formulation of CeNPs failed to provide protection against symptoms and preserve motor function, unless when delivered in conjunction with the immunomodulatory drug lenalidomide (Eitan et al., 2015). This formulation of CeNPs was characterized by a hydrodynamic radius of 34 +/− 6.8 nm (in aqueous solution) (Eitan et al., 2015), a size that may have hindered influx into the brain (brain content of ceria was not presented) and thus may be at least partially responsible for the lack of beneficial biological outcomes in this context. However, the lack of tissue deposition analysis and characterization of ROS levels or damage in the brains of treated mice makes it difficult to pinpoint the precise reason why this other formulation of CeNPs failed to prevent EAE development in this study.

Therefore, to reconcile the observed differential effects of distinct CeNP formulations, we conducted an EAE experiment to test the therapeutic efficacy of several CeNP formulations in parallel. *In vitro* characterization of Cerion NRx custom CeNPs (CNRx CeNPs) (Heckman et al., 2013), Nanophase CeNPs (NP CeNPs), and Treibacher Industrie CeNPs (TI CeNPs) indicated distinct physical characteristics but reasonably similar antioxidant activity for the three types of CeNPs in cell-free systems, ischemic brain tissue, and activated macrophages. However, only the CNRx CeNPs exhibited *in vivo* efficacy, by protecting EAE mice against symptom progression and motor deficits. These observations demonstrate that biological effects observed with one type of CeNP cannot necessarily be generalized to *all* forms of CeNPs and, further, that *in vitro* biological effects may not translate to corresponding effects *in vivo*.

## Materials and Methods

### CeNP Synthesis and Characterization

CNRx CeNPs were synthesized as described (Heckman et al., 2013). TI and NP CeNPs were obtained from Treibacher Industrie (Althofen, Austria; 10% dispersion) and Nanophase Technologies (Romeoville, Illinois), respectively. All materials demonstrated characteristic peaks of (111), (200), (220), and (311) planes of cubic-phased ceria particles. Zeta potentials of the individual particle formulations were determined using a Malvern Zetasizer nano-ZS (Worcestershire, UK). Particle size was measured by DLS (data not shown).

### Antioxidant Enzyme-Mimetic Activity of CeNPs

Catalase-mimetic activity of CeNPs was assessed using the Amplex Red Catalase Assay kit (ThermoFisher Scientific; Grand Island, NY), as reported previously (DeCoteau et al., 2016). Briefly, for measuring catalase-mimetic activity, 60 µM CNRx, TI, or NP CeNPs were incubated with 10 µM H_2_O_2_ in individual wells of black 96-well plates with clear optical bottoms (ThermoFisher Scientific; Grand Island, NY) for 30 minutes. Amplex^®^ Red reagent mix was added to each well and incubated for another 30 minutes. In the presence of horseradish peroxidase, the Amplex^®^ reagent reacts with any unused H_2_O_2_ to generate a fluorescent product, resorufin. Hence, the level of fluorescence at the end of the experiment was inversely related to the catalase activity present in the sample. Fluorescence was measured with a Synergy HT microplate reader (BioTek Instruments; Winooski, VT) using 530/25 nm excitation and 590/35 nm emission filter sets. Samples were assayed in triplicate and in at least three separate experiments. To calibrate the catalase activity of our samples, fluorescence in treatment wells was compared to the fluorescence from a standard curve generated with known concentrations of catalase.

SOD-mimetic activity was measured using a colorimetric SOD activity kit (Enzo Life Sciences; Farmingdale, NY) following the manufacturer's protocol. Briefly, a range of CeNP concentrations (6 µM–5 mM) was added to individual wells of a clear, uncoated microtiter plate. A master-mix containing xanthine oxidase and WST-1 reagent was then added to each well. This was immediately followed by the addition of xanthine to initiate the production of superoxide. Superoxide produced in the reaction can then convert the WST-1 reagent into WST-1 formazan which absorbs light at 450 nm. The absorbance at 450 nm was measured every minute over 10 minutes using a Synergy HT microplate reader (BioTek Instruments; Winooski, VT). From these values we calculated a rate change in absorbance at each different CeNP concentration and compared it to the rate change in absorbance observed in control wells (no CeNPs) to determine the percent inhibition. Thus, in samples with higher levels of SOD-mimetic activity, there is less superoxide available for converting WST to WST-1 formazan, and therefore these samples show a slower rate of absorbance change over ten minutes compared to control. We calculated the concentration of each type of CeNP that was equivalent to a 50% inhibition in the rate of absorbance change, which is equivalent to 1 unit of SOD activity. Samples were assayed in triplicate and at least three separate experiments.

### *Ex Vivo* Hippocampal Ischemia Model of Oxidative Stress

All protocols involving humane use of animals were approved by and conducted in accordance with standards of the St. Lawrence University Institutional Animal Care and Use Committee (#SU 11-12-1, S11-11, and SU 12-10). Harvest of hippocampal brain slices from healthy CD1 mice, subsequent ischemic exposure, and quantification of oxidative stress have been previously described (Estevez et al., 2019). SYTOX Green (Molecular Probes, Invitrogen; Eugene OR) was used to stain dead cells as described (DeCoteau et al., 2016). For these experiments, the brain slices were exposed to ischemic conditions simultaneously with exposure to 5.8 μM CNRx, TI, or NP CeNPs. Artificial cerebrospinal fluid was used as the vehicle (Estevez et al., 2019). The staining of treated samples is expressed as a percentage of staining of control samples; a percentage less than 100% indicates greater viability vs control.

### RAW264.7 Cell Activation and ROS Generation

RAW264.7 cells were provided by Dr. Larry Pease at the Mayo Clinic and were maintained in DMEM media with 10% fetal bovine serum, L-glutamine, and antibiotics/antimycotics. Cells were plated at 1.5 × 10^6^ cells/ml in a black 96-well plate and incubated at 37° Celsius overnight. Lipopolysaccharide (1 µg/ml; Sigma-Aldrich St. Louis, MO) was used to activate the cells and was delivered with or without 5 µM CNRx, TI, or NP CeNPs in cell culture media for 6 hours. Control cells were treated with lipopolysaccharide as well as cell culture media instead of CeNPs. Twenty minutes before the end of incubation, 1 µM H_2_CM-DCFDA dye (Molecular Probes, Invitrogen) was added to each well and the plate was returned to 37°. At the 6 hour time point, the cells were washed with PBS and the fluorescence was then read with the Synergy HT microplate reader (BioTek Instruments; Winooski, VT) using 485/20 nm excitation and 528/20 nm emission filter sets.

### Mice and EAE Disease Induction

Female SJL/J mice purchased from Jackson Laboratories (Bar Harbor, ME) were induced with EAE at 8–10 weeks of age by subcutaneous flank injection of a homogenized mixture of 200 µg PLP 139-151 (Genscript, Piscataway NJ) in 50 µl PBS with 50 µl complete Freund's adjuvant (Sigma-Aldrich) on day 0. An intraperitoneal injection of 200 ng pertussis toxin (List Biological Laboratories, Campbell CA) in 100 µl PBS was also delivered on days 0 and 2.

### CeNP Delivery and Disease Assessment

For experiment comparing the preventative and therapeutic CNRx CeNP regimens (**Figure 6**), mice received intravenous CNRx CeNPs in 100 µl sodium citrate buffer (or vehicle control) on days −1, 0, 3, 7, 14, and 21 (preventative regimen) OR days 3, 7, 14, and 21 (therapeutic regimen) relative to disease induction on day 0. Preventative doses were 15 mg/kg on days −1 and 0, followed by 6 mg/kg on remaining days. All therapeutic doses were 6 mg/kg. For experiment comparing the efficacy of the different CeNP formulations (**Figures 7**–**9**), mice received 6 mg/kg CNRx, TI, or NP CeNPs in 100 µl sodium citrate buffer (or vehicle control) on days 3, 7, 14, and 21 *via* intravenous tail vein injection.

For both experiments, clinical scores were assigned by blinded researchers twice per day: 0, normal tail tone and limb movement; 0.5, tail drags when walking but can still curl around observer's finger; 1, tail has no tone and drags when animal walks; 2, impaired or clumsy gait; 2.5, partial paralysis of one or both hind limbs; 3, complete paralysis of one hind limb; 3.5, complete paralysis of both hind limbs; 4, paralysis of one or both front limbs; 5, moribund. Per our Institutional Animal Care and Use Committee standards, mice scoring a 4 OR a 3.5 for greater than 5 days were euthanized.

### Tests of Motor Function

Approximately 1 week pre-induction of EAE, mice were trained to perform on the rotarod, balance beam, and hanging wire tests to obtain baseline values of motor function. At the end of training, performance on the tasks was evaluated to distribute animals with approximately equivalent functional capabilities to the control and treatment groups for subsequent disease induction. These tests were then performed daily by blinded researchers as previously described (Heckman et al., 2013) to track motor function *via* pairwise analysis as EAE progressed.

### CeNP Biodistribution

On day 35, CeNP biodistribution was assessed by harvesting tissues from mice perfused with PBS immediately following euthanization. Liver, spleen, and brain tissues were frozen for subsequent analysis by inductively-coupled mass spectrometry (ICP-MS; Dartmouth College, Hanover, NH).

### ROS Levels in Hippocampal Sections of EAE Mice

EAE was induced as described. Mice were treated with intravenous CNRx CeNPs or vehicle control in a preventative regimen: 15 mg/kg days −1 and 0 followed by 6 mg/kg on days 3, 7, 14, 21, and 28. Five weeks following the final treatment dose, brains were harvested and hippocampal sections were stained with 5 µM CM-H_2_DCFDA and visualized as previously described (Heckman et al., 2013).

### Statistics

In vitro catalase activity was assessed by one-way ANOVA for main effect followed by Dunnet's post-hoc analysis; the comparison of SOD activity between CNRx and NP CeNPs utilized a Student's t-test. Brain tissue sparing by the CeNP treatments was compared by one-way ANOVA, followed by Holm-Sidak test to compare treatments. Sytox Green levels for each CeNP treatment were also individually compared to control treatment by t-test. CeNP-mediated reduction of ROS levels in LPS-treated RAW264.7 cells was assessed by one-way ANOVA for main effect, followed by Tukey's test for pairwise comparisons. Assessment of the preventative vs therapeutic CNRx delivery regimen efficacy was conducted by analyzing mean clinical scores by Friedman's repeated measures ANOVA on ranks (main effect) followed by Holm-Sidak *post hoc* test to compare each treatment to control (**Figure 6**); a t-test was also used to compare treatments (preventative vs therapeutic). Clinical scores were analyzed by Friedman's repeated measures ANOVA on ranks (main effect) followed by Dunnett's test (**Figure 7A**) or Kruskal-Wallis one-way ANOVA followed by Dunn's method to compare each treatment to control (**Figure 7B**). Also in **Figure 7B**, pairwise comparisons of the treatment groups by Holm-Sidak method indicated a significant difference in area under the curve analysis for CNRx vs TI CeNPs (p < 0.05), but not for CNRx vs NP CeNPs or TI vs NP CeNPs (p > 0.05). Day of disease onset (**Figure 7C**) was assessed by factorial ANOVA (main effect) followed by Holm-Sidak comparison of the individual treatment groups vs control. Repeated-measures ANOVA tests were used to compare EAE animal performance on motor tasks (main effect), followed by Holm-Sidak tests for individual comparisons vs control performance or vs CNRx CeNP-treated animal performance. ROS levels in the brains of EAE mice were compared by t-test.

## Results

### CeNP Characterization

**Figure 1:**
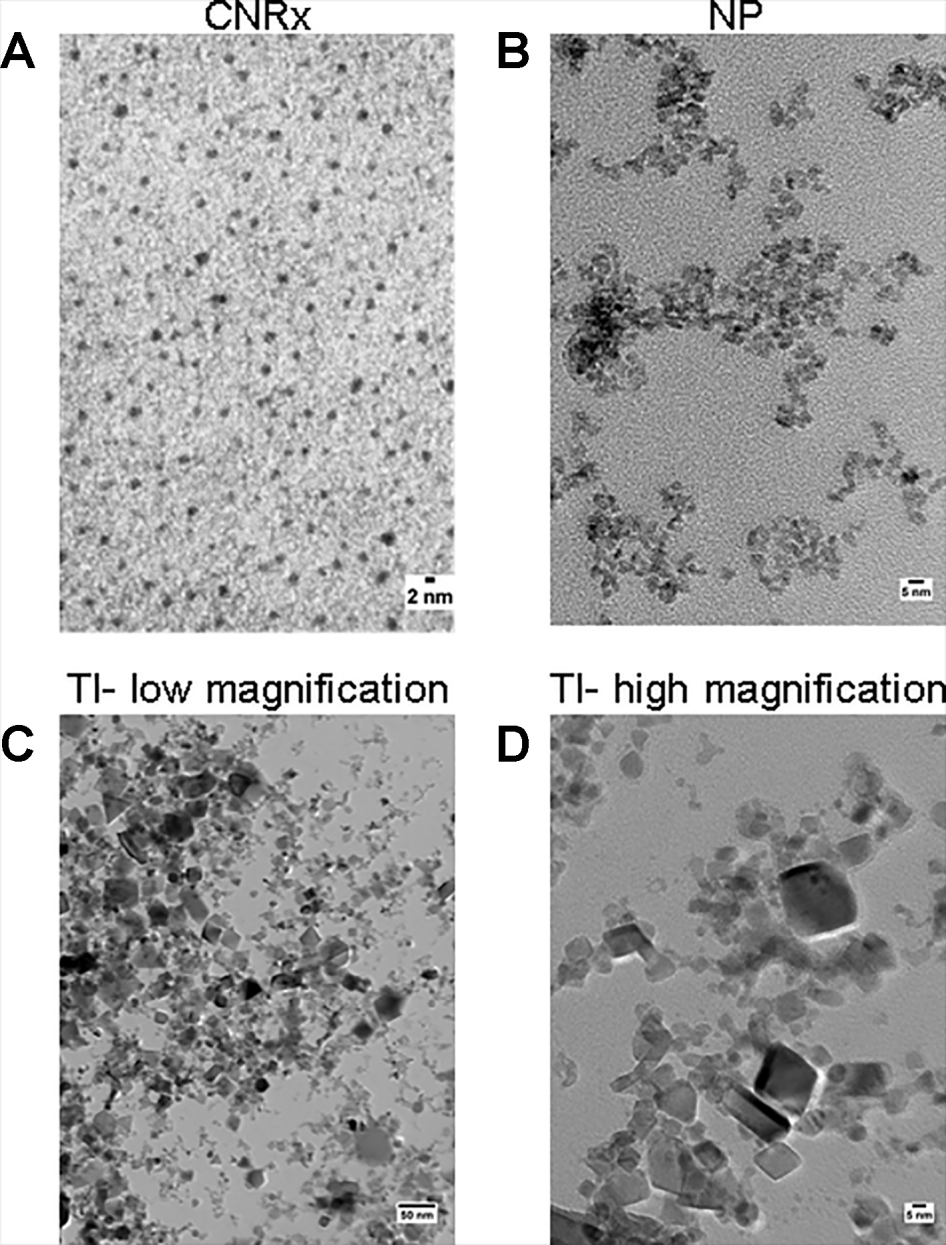
TEM characterization. Cerion NRx (CNRx) **(A)**, Nanophase (NP) **(B)**, and Treibacher Industrie (TI) **(C, D)** cerium oxide nanoparticles (CeNPs) were analyzed by TEM. Low **(C)** and high **(D)** magnification images of the TI CeNPs illustrate the aggregation tendency of this CeNP formulation.

**Figure 2:**
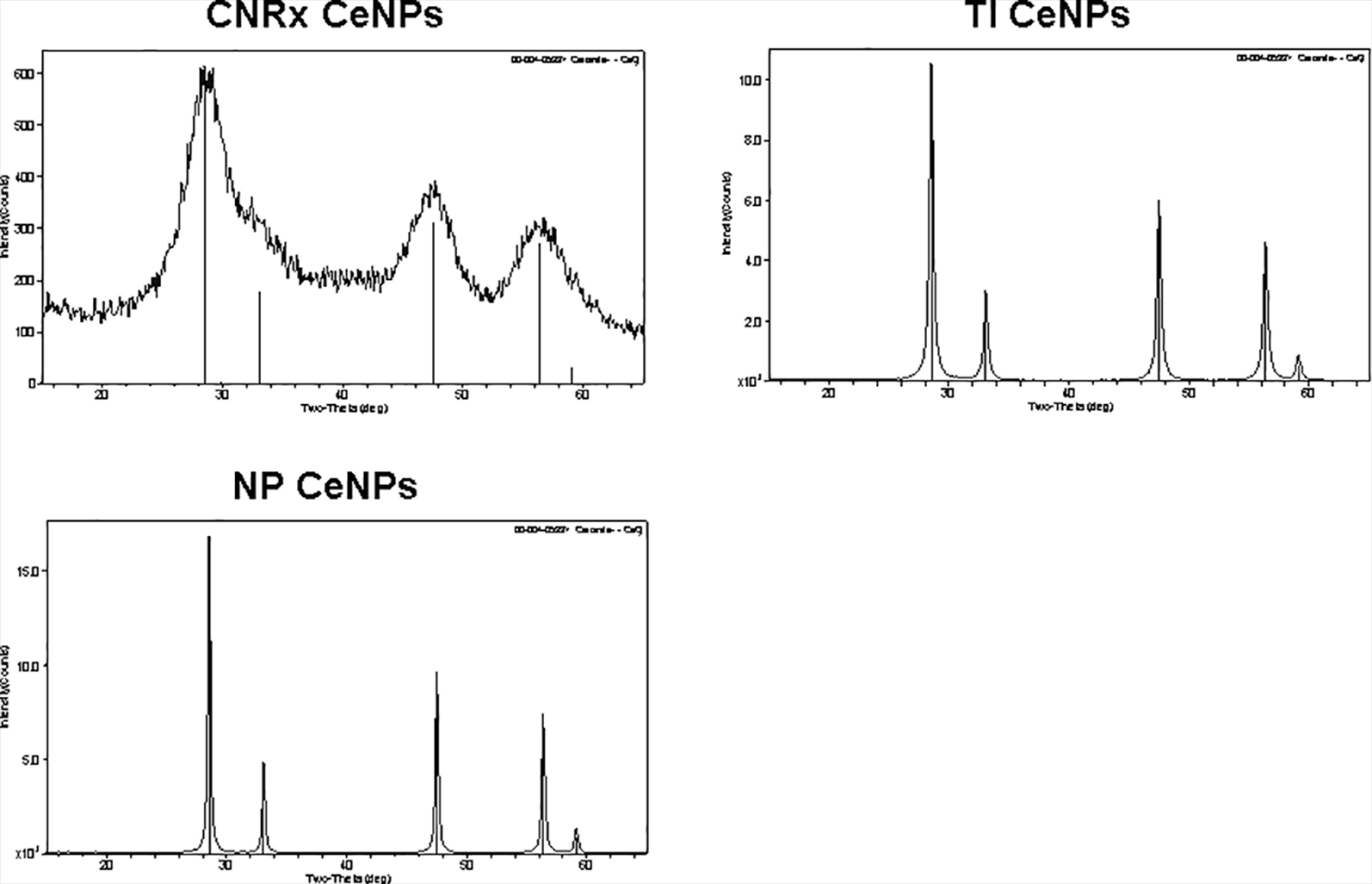
Cerium oxide nanoparticles (CeNPs) crystallinity. Cerion NRx (CNRx), Treibacher Industrie (TI), and Nanophase (NP) CeNPs were assessed by XRD; cerianite reference is shown in each plot as vertical lines.

Cerium oxide nanoparticles synthesized by Cerion NRx (CNRx), Treibacher Industrie (TI), and Nanophase Technologies (NP) were investigated (**Figure 1**). NP CeNPs were synthesized with an acetate stabilizer, while TI and CNRx CeNPs were synthesized with citrate and citrate-EDTA stabilizers, respectively. X-ray diffraction characterization (**Figure 2**) demonstrated a monophasic peak indicative of crystalline ceria for each formulation. Mean diameter of the particles were: CNRx 1.7 ± 0.5 nm, TI 63 ± 15.25 nm (sonicated for three minutes for dispersal prior to analysis), and NP 17.69 ± 5.9 nm (also sonicated). The three formulations had similar zeta potentials: CNRx −23 mV, TI −16 mV, and NP −21 mV.

### CeNP Antioxidant Activity

**Figure 3:**
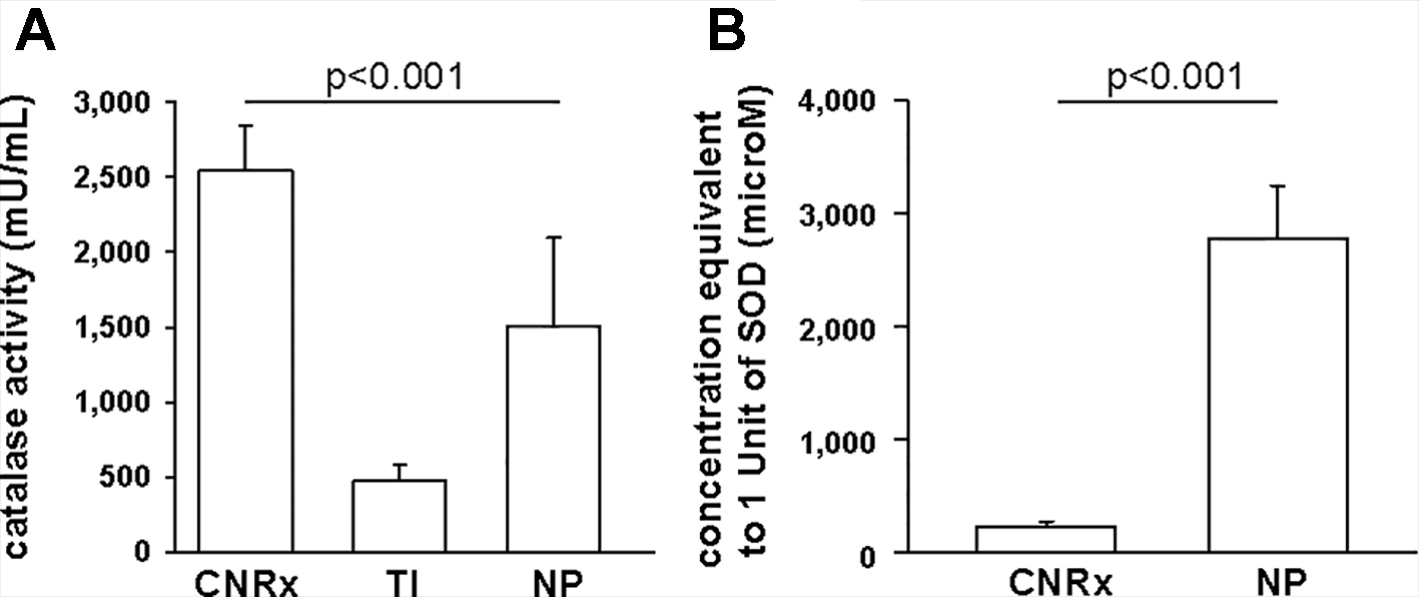
Antioxidant activity of Cerion NRx (CNRx), Treibacher Industrie (TI), and Nanophase (NP) cerium oxide nanoparticles (CeNPs). Cell-free assays were performed to assess relative catalase **(A)** and superoxide dismutase **(B)** activity. **(A)** Catalase-mimetic activity of 60 µM of each type of CeNPs (per reaction) was measured. p < 0.001 among groups (one-way ANOVA for main effect). p < 0.05 CNRx vs each TI, NP (Dunnett's post-hoc analysis). **(B)** CeNP formulations (6 µM–5 mM) were assayed for SOD activity, to find the concentration of each that produced a 50% inhibition in the rate of absorbance change, indicative of 1 unit of superoxide dismutase (SOD) activity. p < 0.001 (Student's t-test). Data are presented as mean ± SD of 4 **(A)** or 3 **(B)** independent experiments.

Since CeNPs have been shown to display potent catalase- and SOD-mimetic activity (Korsvik et al., 2007; Heckert et al., 2008; Pirmohamed et al., 2010), we compared the three CeNP formulations in cell-free assays to estimate enzyme mimetic activity. Identical concentrations of CeNPs yielded varying levels of catalase-mimetic activity (**Figure 3A**) with CNRx CeNPs demonstrating the highest levels (p < 0.001 among groups), approximately 1.7- and 5.3-fold higher than NP and TI, respectively (p< 0.05 CNRx vs TI and NP). The ability of the CeNPs themselves to oxidize the Amplex Red reagent in the absence of H_2_O_2_ was also tested. This oxidase-like activity has been observed at different pH and concentrations (Asati et al., 2009; Lee et al., 2009) and could indicate the propensity of the CeNPs to react undesirably with cellular biomolecules. Interestingly, the oxidase-mimetic activity was negligible, less than 1% of the fluorescence level observed with 10 µM H_2_O_2_, across all three CeNPs tested (data not shown), suggesting a decreased likelihood of general oxidase activity promiscuity, though we did not test other biomolecules. This specific, species dependent activity is consistent with the oxygen vacancy hypothesis of CeNP function (Korsvik et al., 2007; Reed et al., 2014).

To estimate SOD-mimetic activity, a concentration range of each CeNP formulation was tested in order to determine the concentration of each that produced a 50% inhibition in the rate of absorbance change, indicative of 1 unit of SOD activity. Therefore, a lower concentration needed to produce 50% inhibition reflects higher SOD-mimetic activity. CeNRx CeNPs displayed potent SOD-mimetic activity with just 224 ± 50 µM being equivalent to 1 unit of SOD activity (**Figure 3B**). For NP CeNPs, an approximately tenfold higher concentration (2780 ± 464 µM) was needed to produce a 50% inhibition in the rate of absorbance change (p < 0.001). The TI CeNPs were tested up to 3 mM, and a 50% inhibitory concentration could not be reached (data not shown). Higher concentrations could not be tested because there was visible precipitation of the ceria in the wells. Based on this, we conclude that the concentration of TI CeNPs equivalent to 1 unit of SOD activity is greater than 3 mM and that therefore the TI CeNPs have the lowest levels of SOD-mimetic activity of the formulations tested. Thus, similar to another nanoceria formulation that displayed dual catalase and SOD mimetic activity, (Baldim et al., 2018) the CNRx CeNP formulation is shown here to exhibit both antioxidant effects.

### CeNPs Provide Protection Against Ischemic Oxidative Stress in *Ex Vivo* Model

**Figure 4:**
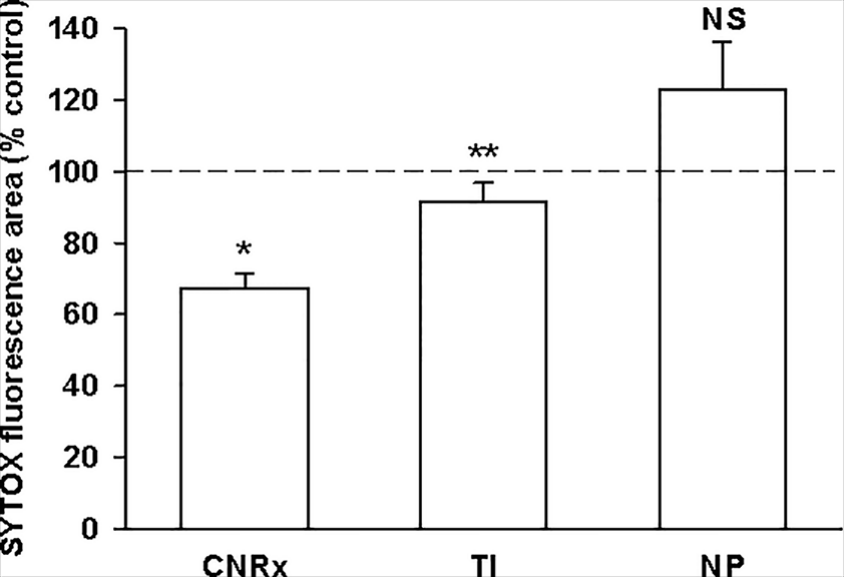
Cerion NRx (CNRx) and Treibacher Industrie (TI) cerium oxide nanoparticles (CeNPs) improve cell viability in brain slices under ischemic stress conditions. Hippocampal brain slices harvested from healthy CD1 mice were exposed to hypoglycemic, acidic, and ischemic conditions for 30 minutes simultaneously with 5.8 µM CNRx, TI, or Nanophase (NP) CeNPs or vehicle (artificial cerebrospinal fluid). Samples were returned to standard non-ischemic conditions and incubated for 24 hours. Cell viability was assessed by SYTOX Green staining. Values less than 100% (control) indicate greater viability. n = 12 age-matched, anatomically paired sections (CNRx), 9 (TI), 7 (NP). CNRx, TI, and NP significantly different than each other p < 0.001 (one-way ANOVA). * p< 0.001 CNRx vs control; ** p = 0.014 TI vs control; NS, not significant NP vs control (Student's t-test).

Exposure of murine brain slices to hypoglycemic and ischemic conditions *ex vivo* induces oxidative stress that triggers cell death and mimics an ischemic stroke (Estevez et al., 2019). Brain sections subjected to ischemic conditions for 30 minutes with or without CeNP treatment were stained with SYTOX Green to quantify cell viability. [(Dead/dying cells stain positive for this fluorescent dye (DeCoteau et al., 2016)]. CNRx and TI CeNP-treated tissues displayed less fluorescence than vehicle-treated control samples, indicated by a % control value less than 100% (**Figure 4**). These values represent a statistically significant improvement in cell viability under ischemic conditions (p < 0.01 CNRx, p = 0.014 TI). Interestingly, in contrast, brain slices exposed to NP CeNPs exhibited more fluorescence than control-treated samples; however this is simply a trend and was not found to be a statistically significant difference (**Figure 4**). Thus, despite displaying antioxidant properties in cell-free systems, not all three types of CeNPs exhibited biological efficacy in this reduced preparation.

### CeNPs Reduce ROS Levels in Activated Macrophages

**Figure 5:**
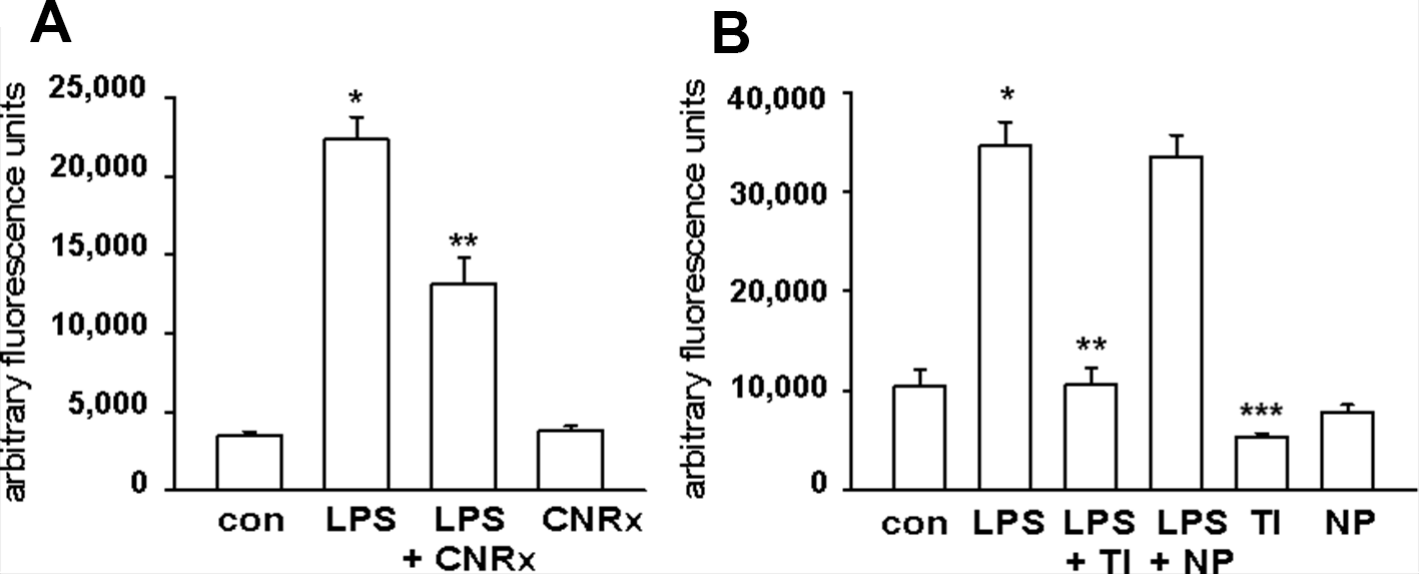
Cerion NRx (CNRx), Treibacher Industrie (TI), and Nanophase (NP) cerium oxide nanoparticles (CeNPs) decrease reactive oxygen species (ROS) levels in activated macrophages. RAW264.7 cells were treated with media as a control (con) or lipopolysaccharide (LPS) with or without 5 μM CNRx **(A)** or 5 μM TI or NP CeNPs **(B)** in cell culture media for 6 hours. The ROS indicator dye H_2_CM-DCFDA was added for the last 20 minutes of incubation, and fluorescence was measured. **(A)** mean +/− SEM of 10–12 samples from four experiments. Main effect: p < 0.001 (one-way ANOVA); * control vs LPS and **LPS vs LPS+CNRx p < 0.05 (Tukey's test). **(B)** mean +/− SEM of 8–9 samples from 3 experiments. LPS main effect p < 0.001 (one-way ANOVA); *control vs LPS and **LPS vs LPS+TI p < 0.05 (Tukey's test). ***control vs TI p < 0.05 (Tukey's test).

Macrophages respond to a range of activating stimuli by generating ROS that are used to kill phagocytosed microbes (Murray and Wynn, 2011) to contribute to innate immunity. Lipopolysaccharide (LPS) is a bacterial component that binds toll-like receptor 4 on macrophages, initiating a pro-inflammatory phenotype (Sanlioglu et al., 2001), and was utilized to induce the production of ROS by the RAW 264.7 murine macrophage cell line. In untreated cells, none of the CeNP formulations had significant pro-oxidant effects, as indicated by similar levels of ROS detected in control samples vs samples exposed to each CeNP alone. LPS induced increased ROS production; these levels were reduced by co-treatment with either CNRx or TI CeNPs (main effect of LPS treatment p < 0.01; both CNRx+LPS and TI+LPS vs LPS alone p < 0.05), but not NP CeNPs (**Figures 5A, B**). TI exposure reduced LPS-induced ROS levels to a greater extent than CNRx CeNPs (~87% vs ~41%, respectively), and even reduced baseline ROS levels in control samples (**Figure 5B**) (main effect of control vs CeNPs alone p < 0.01; control vs TI p < 0.05). Thus, in this context of biologically generated ROS, only CNRx and TI CeNPs demonstrated antioxidant activity in reducing levels of ROS that could be deleterious to cells or tissues in the context of disease.

### CeNPs Differentially Affect Disease Severity in an *in Vivo* Model of Oxidative Stress

**Figure 6:**
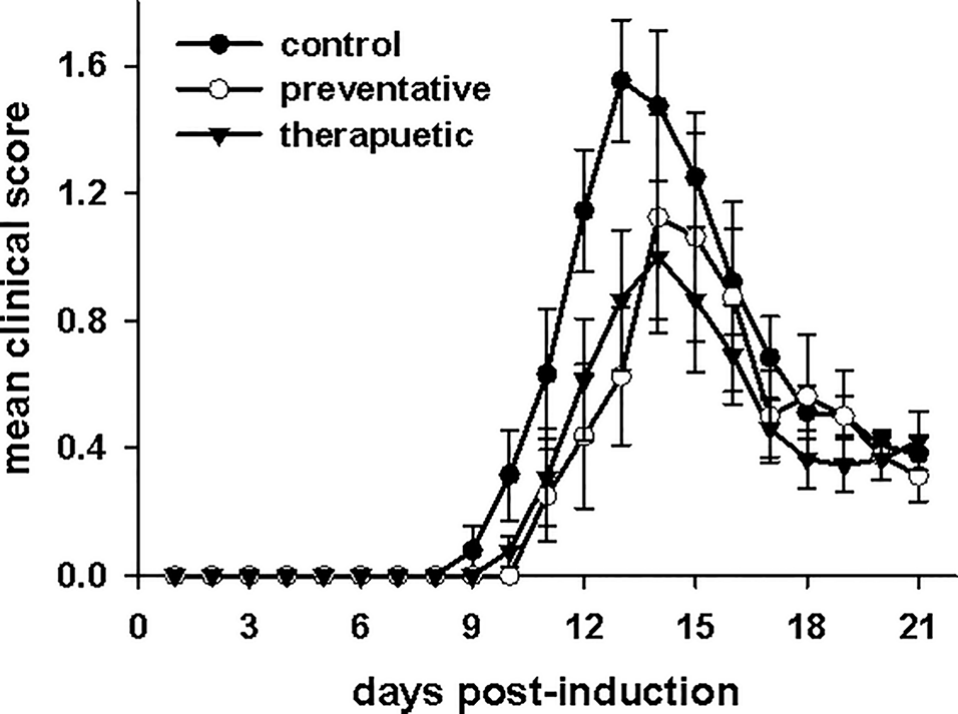
Cerion NRx (CNRx) cerium oxide nanoparticles (CeNPs) lessen disease symptoms in a murine model of multiple sclerosis in both preventative and therapeutic dosage regimens. Female SJL/J mice were induced with experimental autoimmune encephalomyelitis (EAE) and intravenously treated with CNRx CeNPs or vehicle in a preventative dosage (days −1, 0, 3, 7, 14, 21 relative to induction on day 0) or a therapeutic dosage (days 3, 7, 14, 21 relative to induction on day 0). Preventative doses were 15 mg/kg on days−1 and 0, followed by 6 mg/kg on remaining days. All therapeutic doses were 6 mg/kg. Daily mean clinical scores for each group are depicted. The preventative and therapeutic regimens are different than control (p < 0.05, repeated measures ANOVA on Ranks with Holm-Sidak *post hoc* analysis), but are not different from each other (p = 0.658, t-test). n = 8–17 mice per group.

CNRx CeNPs have previously shown efficacy in alleviating the symptoms and motor deficits of mice with the oxidative stress-mediated disease model EAE (Heckman et al., 2013). The EAE model previously tested was a chronic, progressive model of multiple sclerosis (MOG peptide model in C57BL/6 mice), so we wanted to determine whether similar efficacy of CNRx CeNPs was observed in the PLP peptide model of EAE in SJL/J mice, which represents a relapsing/remitting model of multiple sclerosis. A comparison of a preventative CNRx CeNP dosing regimen (beginning day−1 relative to disease induction) and therapeutic CNRx CeNP dosing regimen (beginning day 3 relative to disease induction) was conducted (**Figure 6**). Mean clinical scores for the preventative and therapeutic groups were significantly different than the control group (p < 0.05, repeated measures-ANOVA on ranks with Holm-Sidak *post hoc* analysis), though the clinical scores for the preventative and therapeutic groups were not different from each other (p = 0.658, t-test). The similar efficacy of the preventative and therapeutic regimens led us to utilize the 6 mg/kg CNRx CeNP dose in the therapeutic regimen for our subsequent study comparing different CeNP formulations.

**Figure 7:**
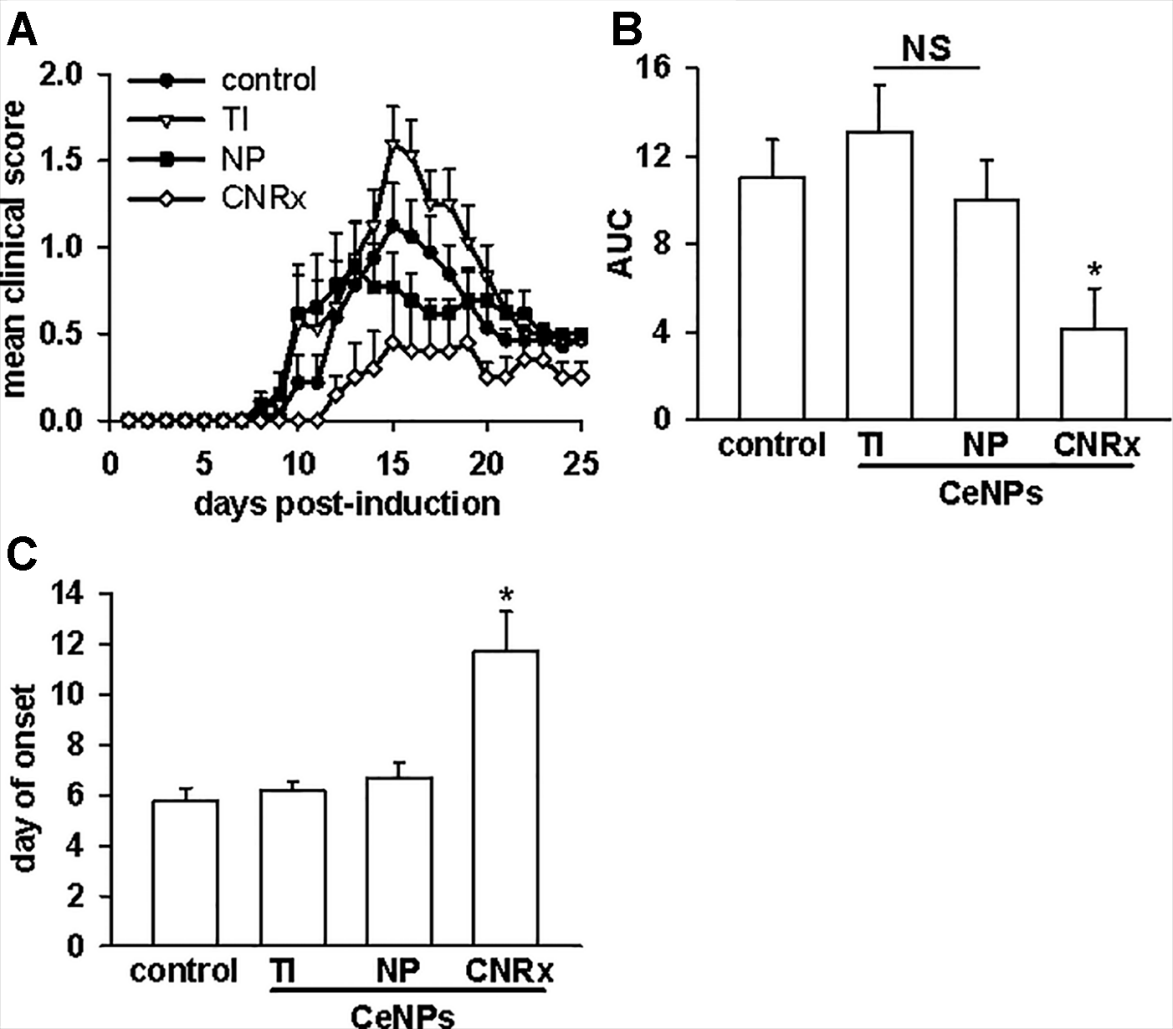
Only Cerion NRx (CNRx) cerium oxide nanoparticles (CeNPs) alleviate disease severity in a murine model of multiple sclerosis. Female SJL/J mice were induced with experimental autoimmune encephalomyelitis (EAE) and intravenously treated with 6 mg/kg Treibacher Industrie (TI), Nanophase (NP), or CNRx CeNPs or vehicle on days 3, 7, 14, 21, and 28 post-induction. **(A)** Daily mean clinical scores for each group are depicted (main effect: p < 0.001, Friedman's repeated measures ANOVA on Ranks). Area under the curve (AUC) analysis of clinical scores over the disease course **(B)** indicates cumulative disease severity (main effect: p = 0.017, Kruskal-Wallis One-Way ANOVA; *CNRx vs control p < 0.05, Dunn's Method). **(C)** Mean day of symptom onset (main effect: p < 0.001, Factorial ANOVA; *CNRx vs control p < 0.001, Holm-Sidak test). n = 10–16 mice per group. NS, not significant.

Given that the TI and NP CeNPs exhibited some degree of antioxidant effects *in vitro*, we hypothesized that these materials could replicate the effects of the CNRx CeNPs in this *in vivo* system. However, when the CeNPs were delivered intravenously to EAE mice on days 3, 7, 14, 21, and 28 post-induction, only the mice treated with CNRx CeNPs exhibited significantly reduced disease severity (p < 0.05) compared to control animals (**Figure 7A**). NP CeNP treatment had no effect (p > 0.05 vs control), though TI CeNP treatment significantly exacerbated disease symptoms in the EAE mice (p < 0.05 vs control) (**Figure 7A**). Area under the curve (AUC) analysis of the mean clinical scores of each group demonstrated that CNRx CeNP-treated animals experienced the most mild cumulative disease severity (p < 0.05 vs control), while TI and NP CeNP treatments failed to provide protection against disease progression (each p > 0.05 vs control) (**Figure 7B**). Similarly, CNRx CeNP treatment delayed the onset of EAE symptoms (p < 0.001 vs control), though TI and NP CeNP treatments did not (each p > 0.05 vs control) (**Figure 7C**).

**Figure 8:**
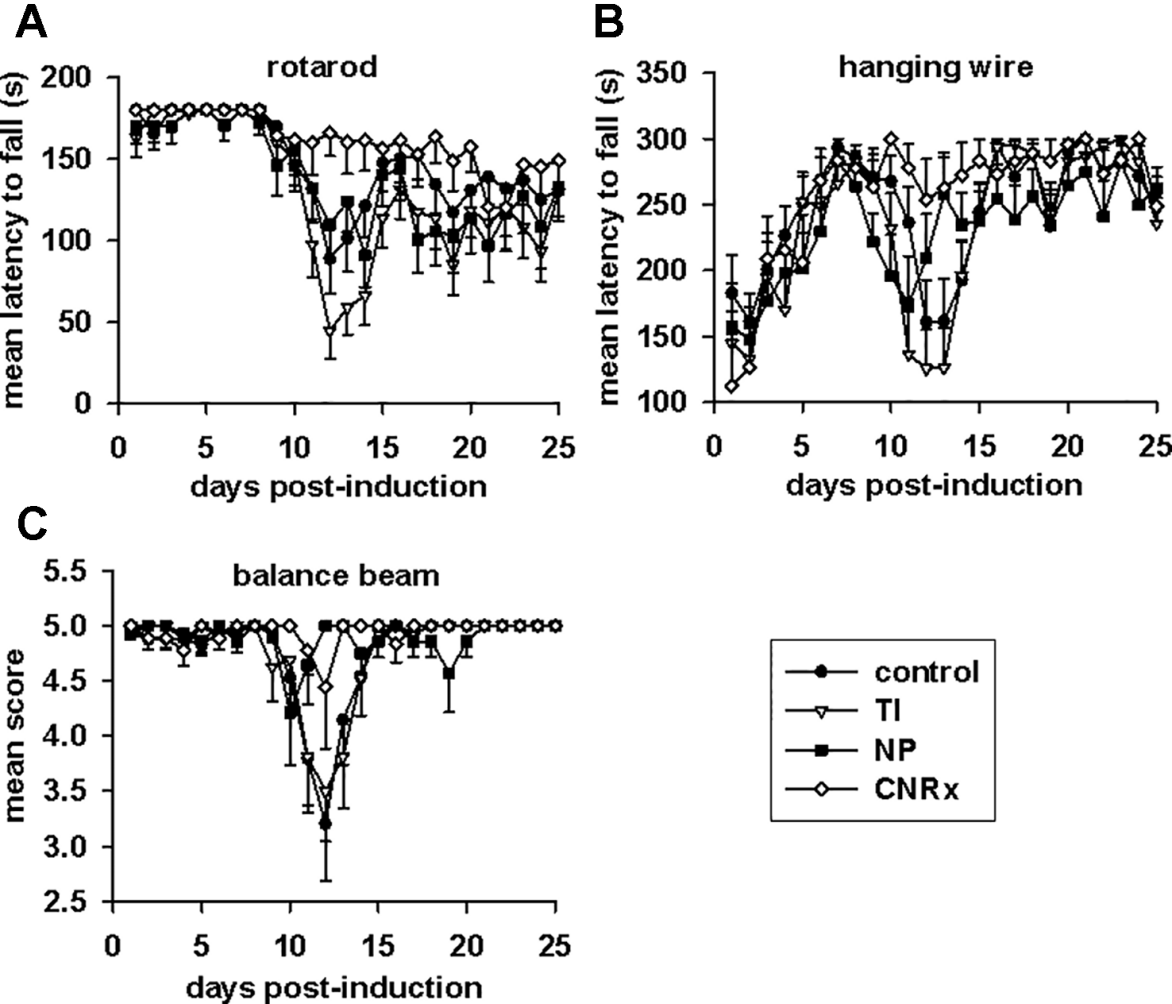
Motor function in experimental autoimmune encephalomyelitis (EAE) mice is preserved by Cerion NRx (CNRx) cerium oxide nanoparticle (CeNP) treatment, but worsened by Treibacher Industrie (TI) and Nanophase (NP) CeNP treatment. EAE mice were tested daily in the rotarod **(A)**, hanging wire **(B)**, and balance beam **(C)** tasks. Higher mean latency to fall from the rotarod and hanging wire and higher mean balance beam score indicate better motor function. See *Results* for statistical comparison. n = 10–16 per group. s, seconds.

Tests of motor capabilities serve to illustrate the functional deficits experienced by EAE animals (Brooks and Dunnett, 2009). The rotarod assesses overall coordination of hind and front limb movement, while the hanging wire test evaluates front limb grip strength (Brooks and Dunnett, 2009). Higher mean latency to fall from these apparatuses is indicative of better motor function. Balance capabilities are measured by the balance beam test (Brooks and Dunnett, 2009), with a higher score also representing less functional deficit. On all three tests (**Figures 8A–C**), there was a significant difference in the performances of control and CeNP-treated animals (main effect: p < 0.001 rotarod; p = 0.001 hanging wire; p = 0.047 balance beam, repeated measures ANOVA). Rotarod performances of TI, NP, and CNRx CeNP-treated mice were significantly different than control mice, but both the TI and NP CeNP-treated animals actually performed worse than control animals (p < 0.05) (**Figure 8A**). (This and all additional individual comparisons of treatment groups vs control or treatment groups vs CNRx CeNP-treated animals in **Figure 8** were made by Holm-Sidak test.) In addition to performing significantly better than control animals (p < 0.05), CNRx CeNP-treated mice also exhibited better rotarod performance than both TI and NP CeNP-treated animals (both comparisons p < 0.001). Again, with the hanging wire task, TI and NP CeNP-treated mice performed worse at various time points (though not significantly, p > 0.05) than control animals (**Figure 8B**). Only the CNRx CeNP-treated mice had better performance overall; this was the only treatment group that demonstrated a significantly different performance than control animals (p = 0.015). Similarly, the performance of control animals on the balance beam was significantly different than that of only the CNRx CeNP-treated mice (p = 0.023); for this motor test, the TI and NP CeNP-treated animals did not perform different than controls (p > 0.05) (**Figure 8C**). These results parallel disease severity (clinical score) results (**Figure 7A**), confirming that CNRx CeNPs exhibit biological efficacy in the EAE model, while the TI and NP CeNPs fail to achieve the same protective results.

### CeNP Formulations Exhibit Differences in Biodistribution Patterns

**Figure 9:**
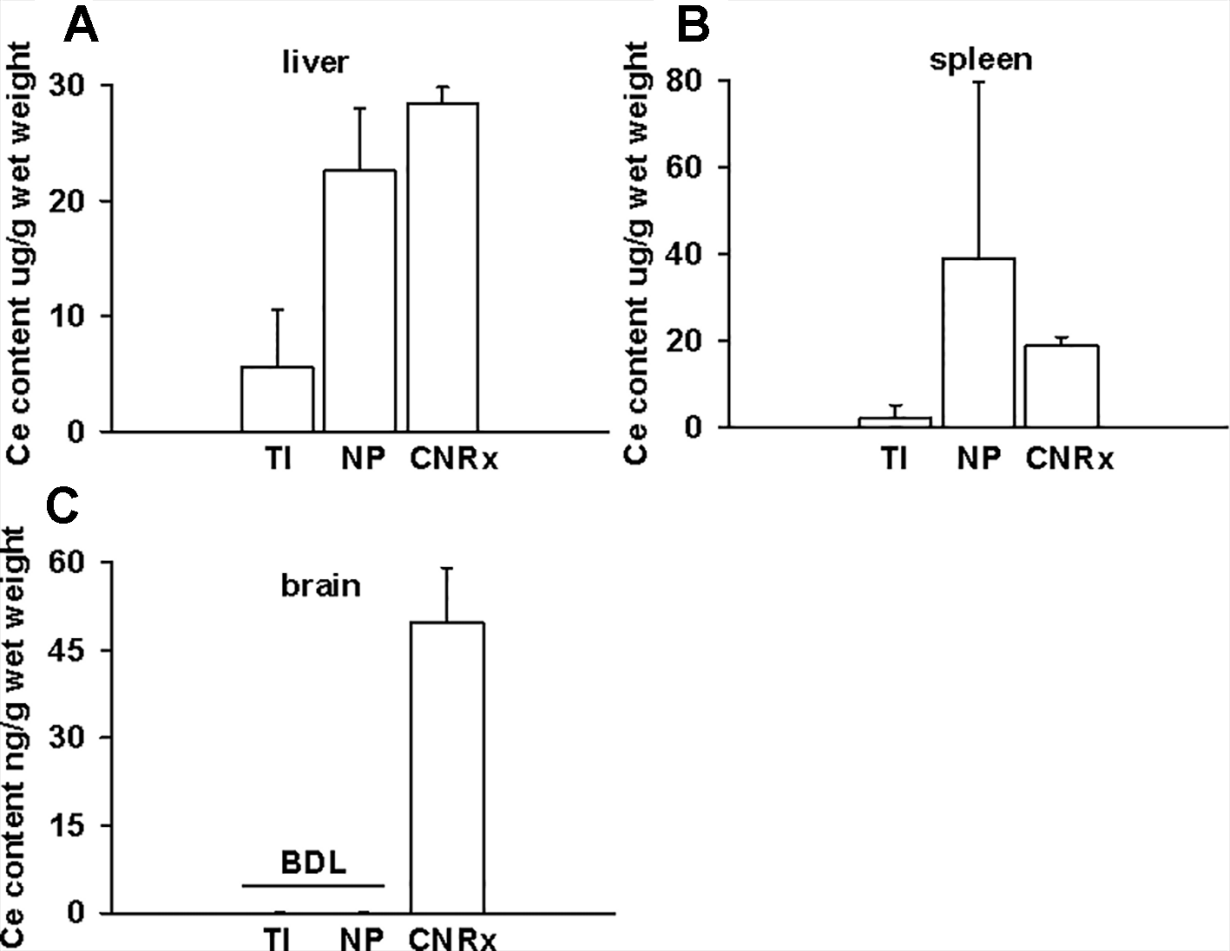
Only Cerion NRx (CNRx) cerium oxide nanoparticles (CeNPs) distribute to the brains of experimental autoimmune encephalomyelitis (EAE) mice. On day 35 post-induction, EAE mice were euthanized and perfused with PBS. Liver **(A)**, spleen **(B)**, and brain **(C)** tissues were harvested, and ceria content was analyzed by ICP-MS. Results are presented as mean + SEM in µg ceria/g wet weight. BDL, below detection limit.

**Figure 10:**
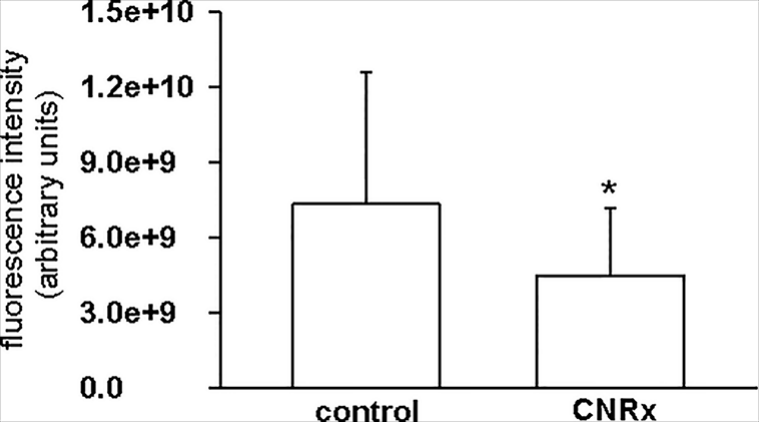
Cerion NRx (CNRx) cerium oxide nanoparticles (CeNPs) exhibit prolonged *in vivo* antioxidant activity. Experimental autoimmune encephalomyelitis (EAE) mice treated with CNRx CeNPs or vehicle control (15 mg/kg days −1 and 0 followed by 6 mg/kg days 3, 7, 14, 21, 28) were euthanized 5 weeks after their final treatment dose. Brains were harvested and reactive oxygen species (ROS) levels were measured by H_2_CM-DCFDA staining. Results represent 20 matched pairs of brain slices from four control and four CNRx treated mice. * p = 0.007 (Student's t-test).

Tissues were also harvested from CeNP-treated EAE mice to determine whether differences in treatment effects could be attributable to ceria deposition. All three types of CeNPs were detected in the liver and spleen, and levels of TI CeNPs were lowest in both tissues (**Figures 9A, B**). Liver deposition was highest for CNRx CeNPs, relative to the other CeNPs, while NP CeNPs exhibited highest distribution to the spleen (despite great variability.) Only CNRx CeNPs were detectable in the brains of treated animals (**Figure 9C**), which parallels the protection against symptoms observed in this treatment group. To observe the functionality of the CNRx CeNPs, brains were harvested from control and CNRx CeNP-treated EAE mice 5 weeks after the final CeNP injection (delivered day 28 post-induction) and stained with a ROS indicator dye. Not only did the CNRx CeNPs deposit in the brain (**Figure 9C**), but they also diminished the level of ROS in the hippocampus of treated EAE mice compared to controls (**Figure 10**). These hippocampal slices included portions of the M1 and M2 motor cortex, which provides an understanding of ROS conditions present in these upper motor neuron pools. Significantly reduced brain levels of ROS suggest that CNRx CeNPs retain their *in vitro* antioxidant activity *in vivo*.

## Discussion

This parallel examination of different CeNP formulations in *in vitro* and *in vivo* assays supports two key conclusions. First, not all CeNP formulations have identical biological activity and second, *in vitro* assays are insufficient to reliably predict functional activity in a whole organism.

Though custom CNRx CeNPs provided protection against the murine model of multiple sclerosis, both TI and NP CeNPs failed to lessen (or even exacerbated) disease severity (**Figure 7**). Characterization of these CeNPs suggests parameters potentially responsible for these variable *in vivo* effects. Differences in particle size, zeta potential, stabilization coating, or a combination of these and other variables may influence the biological identity of the CeNPs. Larger particulate CeNPs (He et al., 2010) or CeNP aggregates have an increased likelihood of phagocytic uptake, which could be responsible for disproportionate clearance of NP and TI CeNPs (17.69 and 63 nm, respectively versus 1.5 nm for CNRx CeNPs), and thus less available therapeutic material. In a direct comparison of differently sized CeNPs, a greater percentage of total administered small (5 nm) citrate-stabilized intravenously administered CeNPs deposited in the liver and spleen compared to similarly stabilized larger CeNPs (55 nm) (Yokel et al., 2013). Smaller nanoparticles can also reach more diverse tissues when delivered intravenously, whereas larger, gold nanoparticles distribute primarily to the blood, liver, and spleen (De Jong et al., 2008). The observed deposition of the relatively small CNRx CeNPs to the brain tissue is congruent with this distribution principle. Importantly, the CNRx CeNPs reach the brain of healthy mice after intravenous delivery (Heckman et al., 2013), though other CeNPs (citrate-stabilized) ranging from 5 to 55 nm in size (non-hydrated) failed to reach the brain in appreciable levels in healthy rats (Yokel et al., 2013). Though minute levels of the citrate-stabilized CeNPs were detected in the brain in this study (Yokel et al., 2013), the tissue was not perfused prior to analysis, suggesting that some or all of the detected ceria could be present in the residual blood/vasculature. Though the authors could not find definitive evidence of this, it seems unlikely that the larger particles tested (30 and 55 nm) could have crossed the blood brain barrier (BBB) (*via* diffusion or paracellular transport), which excludes materials larger than 3 nm when intact (Komarova et al., 2017). In the context of EAE, treatment efficacy is contingent upon the ability of the CeNPs to cross the BBB. Not surprisingly, even taking into consideration the disrupted BBB characteristic of EAE disease (Stohl et al., 1979; Linthicum et al., 1982; Claudio et al., 1990), the relatively small CNRx CeNPs were detected in the highest levels in brain tissue (**Figure 9**), an observation that correlates with best protection against EAE severity (**Figure 7**) and reduction of hippocampal ROS levels (**Figure 10**). Even with a disrupted BBB, no detectable amounts of NP and TI CeNPs were able to reach this location.

The observed differences in CNRx, TI, and NP CeNP efficacy is in line with previous findings for CeNP-based EAE treatment: monodispersed CNRx CeNPs (hydrodynamic size = 2.9 nm) provide protection (Heckman et al., 2013) while other CeNPs (hydrodynamic size ~ 34 nm) do not (Eitan et al., 2015). Certainly, size is not the only variable distinct between the CeNPs used in these previous studies or in the current study, but it does influence the quantity and identify of proteins that bind to form a protein corona, as an independent factor (Tenzer et al., 2011) or when varied with another nanoparticle characteristic such as surface modifications (Lundqvist et al., 2008). Given the unique stabilization and size of the CNRx CeNPs, they likely bind a suite of distinct molecules that theoretically can alter the distribution profile of the nanoparticle. For example, the adherence of albumin and ApoE to the CNRx CeNPs (Heckman et al., 2014) is thought to support their deposition in the brain. Though beyond the scope of this study, characterization of the protein corona of the TI and NP CeNPs could reveal differences in the quantity and identity of adsorbed proteins responsible for differential tissue distribution, and even biological efficacy. A nanoparticle's zeta potential can also influence the composition of the protein corona, as this characteristic also affects the amount of protein adsorption (Patil et al., 2007). Though the TI, NP, and CNRx CeNPs have similar negative zeta potentials, this parameter must be considered along with size in predicting tissue uptake and biological activity.

Nanoparticle coating or stabilization is typically utilized to achieve reduced reticuloendothelial uptake (Murugan et al., 2015), direct the nanoparticle to a particular tissue target (Cimini et al., 2012; Hijaz et al., 2016), or to provide a functional property such as reduced angiogenesis (Lord et al., 2013). Minor changes to surface chemistry, for example by varying the density of polyethylene glycol coating, yield distinct profiles of corona proteins and differing macrophage uptake patterns (Walkey et al., 2012). The CeNPs utilized in this study are also distinct in their stabilization material. NP CeNPs are stabilized with acetate, whereas TI and CNRx CeNPS are stabilized with citrate and citrate/EDTA, respectively. These stabilizers are not purposefully chosen in order to evade reticuloendothelial uptake or direct nanoparticle distribution, though the differing distribution patterns of the three types of CeNPs suggests that the acetate and citrate-alone stabilizers may pre-dispose (or at least not prevent) uptake by phagocytic cells. TI and NP CeNPs were detected predominantly in the liver and spleen (**Figure 8**), consistent with distribution of other citrate stabilized CeNPs (5–55 nm, non-hydrated) to these tissues in healthy animals (Yokel et al., 2013). Even with variability in the extent of citrate coating (15–40%), these CeNPs reached the brain in similar low levels (Yokel et al., 2013), suggesting that citrate alone stabilization may not support physicochemical properties appropriate for brain distribution. In contrast, the citrate-EDTA coated CeNPs distributed not only to the liver and spleen but also to the brain, suggesting that this unique coating combination enables the relatively long half-life of the CNRx CeNPs in the bloodstream (4 hours) (Heckman et al., 2013) [compared to minutes with other CeNPs (Yokel et al., 2009)] that lengthens the timeframe possible for tissue distribution. The stabilizer coating of the tested nanoparticles is also likely responsible for their propensity to aggregate. The CNRx CeNPs remain monodispersed over time (Heckman et al., 2013), while the TI and NP CeNPs precipitate out of solution, creating chalk-like mixtures of material *in vitro* (data not shown). The aqueous nature of *in vivo* biological systems likely supports this aggregation that, as noted, affects the cellular uptake, corona formation, and biodistribution of nanomaterials. A more systematic study separately varying the individual characteristics of CeNPs will be necessary to elucidate which is most responsible for variable *in vivo* effects. In particular, synthesizing all three CeNPs with the same stabilizer or equilibrating the size of all three CeNPs by making larger CNRx CeNPs or smaller TI and NP CeNPs would aid in clarifying which characteristic is most relevant for these biological outcomes. In other experiments, we have demonstrated that the stabilizer dictates biological effects of CNRx CeNPs (Estevez et al., 2019), though these different CeNPs were not tested in the *in vivo* EAE model.

After observing antioxidant activity from all three types of CeNPs in a range of *in vitro* assays, we expected to observe similar efficacy in the oxidative stress EAE disease model. While all three types of CeNPs demonstrated antioxidant activity *in vitro* in at least one of the cell-free, cell line and *ex vivo* tissue assays performed (**Figures 3**–**5**), only the CNRx CeNPs yielded protection against an *in vivo* disease model of oxidative stress, EAE (**Figures 7** and **8**). We hypothesize that the differential tissue distribution of the CeNP formulations is responsible for these distinct *in vivo* effects, based upon the improved EAE outcomes in animals treated with CNRx CeNPs, which exhibited the highest localization to the brain compared to the other formulations. It is important to emphasize that the absence of the other CeNPs from the brain does not mean that they are not without biological effects in the context of EAE. For example, relatively low levels of the TI CeNPs were observed collectively from the brain, spleen, and liver of EAE mice, and these animals also displayed exacerbated disease. This set of results could suggest that the TI CeNPs are having biological effects in the periphery (such as induction of cytokine release) that can influence events in the CNS. We have not explored whether such peripheral effects of any of the CeNP formulations tested here actually exist or influence the pathology of EAE disease.

These results provide specific cautionary support for the notion that *in vitro* systems are inadequate for the prediction of how nanomaterials will behave in *in vivo* models (Fischer and Chan, 2007). In this study, not only is toxicity a consideration, but also the biological efficacy of the CeNPs' inherent redox properties. Though none of the CeNPs tested here induced detectable toxicity either *in vivo* or *in vitro*, the discrepancy between measured antioxidant activity *in vitro* and functional activity *in vivo* illustrates the difficulty in simply extrapolating *in vivo* efficacy from results observed *in vitro*. Importantly, administration of nanomaterials to an intact organism must take into consideration not only the original protein corona formed upon introduction, but also the evolution of the constituents of the protein corona as the nanomaterial progresses through different biological compartments. *In vitro* assays, even those with intact tissues (i.e. brain slices), cannot replicate the native biological milieu; their predictive power is predicated on their ability to recapitulate the *in vivo* condition. This can only be achieved by comparing the performance of the material in both test beds first. Once the correlative nature of the relationship has been established, the *in vitro* preparation can be adopted as an appropriate translational screening tool.

In summary, these results suggest that favorable characteristics for *in vivo* CeNP antioxidant efficacy in the CNS include: small size, monodispersity, and stabilization with citrate-EDTA. Further study is necessary to understand the role of each of these characteristics in achieving tissue distribution while maintaining redox capabilities and whether they are exclusive or interdependent upon each other. In pursuit of this understanding, inevitably, *in vitro* assays will be performed to screen newly synthesized materials. However, this study illustrates quite clearly that this analysis provides only part of the picture for CeNPs and that *in vivo* assessment of efficacy should be added to the list of “screening” assays if the material is intended for whole organism application.

## Data Availability Statement

The datasets generated for this study are available on request to the corresponding author.

## Ethics Statement

The animal study was reviewed and approved by the St. Lawrence University Institutional Animal Care and Use Committee.

## Author Contributions

KH, AE, WD, and JE designed experiments. KH, AE, WD, JE, and BH-E analyzed and interpreted data. KH, AE, WD, JE, BH-E, JC, SV, and SR conducted experiments. KH, AE, and JE wrote the manuscript.

## Funding

Portions of KH, JE, AE, and WD work has been funded by Cerion NRx, Rochester, New York. Remaining funding provided by St. Lawrence University, Canton, NY.

## Conflict of Interest

JE, AE, and WD are minority shareholders in Cerion NRx.

The remaining authors declare that the research was conducted in the absence of any commercial or financial relationships that could be construed as a potential conflict of interest.

## References

[B1] Asati A. Santra S. Kaittanis C. Nath S. Perez J. M. (2009). Oxidase-like activity of polymer-coated cerium oxide nanoparticles. Angew. Chem. Int. Ed. Engl. 48 (13), 2308–2312. 10.1002/anie.200805279 19130532 PMC2923475

[B2] Bailey Z. S. Nilson E. Bates J. A. Oyalowo A. Hockey K. S. Sajja V. S. (2016). Cerium oxide nanoparticles improve outcome after in vitro and in vivo mild traumatic brain injury. J. Neurotrauma. 33, 1–11. 10.1089/neu.2016.4644 27733104 PMC7249477

[B49] Baldim V. Bedioui F. Mignet N. Margaill I. Berret J. -F (2018). The enzyme-like catalytic activity of cerium oxide nanoparticles and its dependency on Ce3+ surface area concentration. Nanoscale 10, 6971–6980. 10.1039/C8NR00325D 29610821

[B3] Brooks S. P. Dunnett S. B. (2009). Tests to assess motor phenotype in mice: a user's guide. Nat. Rev. Neurosci. 10 (7), 519–529. 10.1038/nrn2652 19513088

[B4] Cimini A. D'Angelo B. Das S. Gentile R. Benedetti E. Singh V. (2012). Antibody-conjugated PEGylated cerium oxide nanoparticles for specific targeting of Aβ aggregates modulate neuronal survival pathways. Acta Biomater. 8 (6), 2056–2067. 10.1016/j.actbio.2012.01.035 22343002

[B5] Claudio L. Kress Y. Factor J. Brosnan C. F. (1990). Mechanisms of edema formation in experimental autoimmune encephalomyelitis. The contribution of inflammatory cells. Am. J. Pathol. 137 (5), 1033–1045. 2240157 PMC1877669

[B6] De Jong W. H. Hagens W. I. Krystek P. Burger M. C. Sips A. J. Geertsma R. E. (2008). Particle size-dependent organ distribution of gold nanoparticles after intravenous administration. Biomaterials 29 (12), 1912–1919. 10.1016/j.biomaterials.2007.12.037 18242692

[B7] DeCoteau W. Heckman K. L. Estevez A. Y. Reed K. J. Costanzo W. Sandford D. (2016). Cerium oxide nanoparticles with antioxidant properties ameliorate strength and prolong life in mouse model of amyotrophic lateral sclerosis. Nanomedicine 12 (8), 2311–2320. 10.1016/j.nano.2016.06.009 27389143

[B8] Dowding J. M. Dosani T. Kumar A. Seal S. Self W. T. (2012). Cerium oxide nanoparticles scavenge nitric oxide radical (˙NO). Chem. Commun. (Camb) 48 (40), 4896–4898. 10.1039/c2cc30485f 22498787

[B9] Dowding J. M. Seal S. Self W. T. (2013). Cerium oxide nanoparticles accelerate the decay of peroxynitrite (ONOO(-)). Drug Delivery Transl. Res. 3 (4), 375–379. 10.1007/s13346-013-0136-0 PMC3736600 23936755

[B10] Dunnick K. M. Pillai R. Pisane K. L. Stefaniak A. B. Sabolsky E. M. Leonard S. S. (2015). The effect of cerium oxide nanoparticle valence state on reactive oxygen species and toxicity. Biol. Trace Elem. Res. 166 (1), 96–107. 10.1007/s12011-015-0297-4 25778836 PMC4469090

[B11] Eitan E. Hutchison E. R. Greig N. H. Tweedie D. Celik H. Ghosh S. (2015). Combination therapy with lenalidomide and nanoceria ameliorates CNS autoimmunity. Exp. Neurol. 273, 151–160. 10.1016/j.expneurol.2015.08.008 26277686 PMC4644463

[B48] Estevez A. Y. Ganesana M. Trentini J. F. Olson J. E. Li G. Boateng Y. O. (2019). Antioxidant enzyme-mimetic activity and neuroprotective effects of cerium oxide nanoparticles stablized with various ratios of citric acid and EDTA. Biomolecules 9, E562. 10.3390/biom9100562 31623336 PMC6843313

[B13] Fischer H. C. Chan W. C. (2007). Nanotoxicity: the growing need for in vivo study. Curr. Opin. Biotechnol. 18 (6), 565–571. 10.1016/j.copbio.2007.11.008 18160274

[B14] Hardas S. S. Butterfield D. A. Sultana R. Tseng M. T. Dan M. Florence R. L. (2010). Brain distribution and toxicological evaluation of a systemically delivered engineered nanoscale ceria. Toxicol. Sci. 116 (2), 562–576. 10.1093/toxsci/kfq137 20457660

[B15] He C. Hu Y. Yin L. Tang C. Yin C. (2010). Effects of particle size and surface charge on cellular uptake and biodistribution of polymeric nanoparticles. Biomaterials 31 (13), 3657–3666. 10.1016/j.biomaterials.2010.01.065 20138662

[B16] Heckert E. G. Karakoti A. S. Seal S. Self W. T. (2008). The role of cerium redox state in the SOD mimetic activity of nanoceria. Biomaterials 29 (18), 2705–2709. 10.1016/j.biomaterials.2008.03.014 18395249 PMC2396488

[B17] Heckman K. L. Decoteau W. Estevez A. Reed K. J. Costanzo W. Sanford D. (2013). Custom cerium oxide nanoparticles protect against a free radical mediated autoimmune degenerative disease in the brain. ACS Nano. 7, 10582–10596. 10.1021/nn403743b 24266731

[B18] Heckman K. L. Erlichman J. Reed K. Skeels M. (2014). Application of mass spectrometry to characterize localization and efficacy of nanoceria in vivo. Adv. Exp. Med. Biol. 806, 561–579. 10.1007/978-3-319-06068-2_28 24952203

[B19] Hijaz M. Das S. Mert I. Gupta A. Al-Wahab Z. Tebbe C. (2016). Folic acid tagged nanoceria as a novel therapeutic agent in ovarian cancer. BMC Cancer 16, 220. 10.1186/s12885-016-2206-4 26979107 PMC4791781

[B20] Hirst S. M. Karakoti A. Singh S. Self W. Tyler R. Seal S. (2013). Bio-distribution and in vivo antioxidant effects of cerium oxide nanoparticles in mice. Environ. Toxicol. 28 (2), 107–118. 10.1002/tox.20704 21618676

[B21] Komarova Y. A. Kruse K. Mehta D. Malik A. B. (2017). Protein interactions at endothelial junctions and signaling mechanisms regulating endothelial permeability. Circ. Res. 120 (1), 179–206. 10.1161/CIRCRESAHA.116.306534 28057793 PMC5225667

[B22] Korsvik C. Patil S. Seal S. Self W. T. (2007). Superoxide dismutase mimetic properties exhibited by vacancy engineered ceria nanoparticles. Chem. Commun. (Camb) (10), 1056–1058. 10.1039/b615134e 17325804

[B23] Kwon H. J. Cha M. Y. Kim D. Kim D. K. Soh M. Shin K. (2016). Mitochondria-targeting ceria nanoparticles as antioxidants for alzheimer's disease. ACS Nano. 10 (2), 2860–2870. 10.1021/acsnano.5b08045 26844592

[B24] Lee C. W. Chen Y. C. Ostafin A. (2009). The accuracy of Amplex Red assay for hydrogen peroxide in the presence of nanoparticles. J. BioMed. Nanotechnol. 5 (5), 477–485. 10.1166/jbn.2009.1055 20201421

[B25] Lee S. S. Song W. Cho M. Puppala H. L. Nguyen P. Zhu H. (2013). Antioxidant properties of cerium oxide nanocrystals as a function of nanocrystal diameter and surface coating. ACS Nano. 7 (11), 9693–9703. 10.1021/nn4026806 24079896

[B26] Linthicum D. S. Munoz J. J. Blaskett A. (1982). Acute experimental autoimmune encephalomyelitis in mice. I. Adjuvant action of Bordetella pertussis is due to vasoactive amine sensitization and increased vascular permeability of the central nervous system. Cell Immunol. 73 (2), 299–310. 10.1016/0008-8749(82)90457-9 6891621

[B27] Lord M. S. Tsoi B. Gunawan C. Teoh W. Y. Amal R. Whitelock J. M. (2013). Anti-angiogenic activity of heparin functionalised cerium oxide nanoparticles. Biomaterials 34 (34), 8808–8818. 10.1016/j.biomaterials.2013.07.083 23942211

[B28] Lundqvist M. Stigler J. Elia G. Lynch I. Cedervall T. Dawson K. A. (2008). Nanoparticle size and surface properties determine the protein corona with possible implications for biological impacts. Proc. Natl. Acad. Sci. U.S.A. 105 (38), 14265–14270. 10.1073/pnas.0805135105 18809927 PMC2567179

[B29] Manne N. D. Arvapalli R. Nepal N. Shokuhfar T. Rice K. M. Asano S. (2015a). Cerium oxide nanoparticles attenuate acute kidney injury induced by intra-abdominal infection in Sprague-Dawley rats. J. Nanobiotechnol. 13, 75. 10.1186/s12951-015-0135-z PMC4619421 26498824

[B30] Manne N. D. Arvapalli R. Nepal N. Thulluri S. Selvaraj V. Shokuhfar T. (2015b). Therapeutic potential of cerium oxide nanoparticles for the treatment of peritonitis induced by polymicrobial insult in Sprague-Dawley rats. Crit. Care Med. 43 (11), e477–e489. 10.1097/CCM.0000000000001258 26327202

[B31] Monopoli M. P. Aberg C. Salvati A. Dawson K. A. (2012). Biomolecular coronas provide the biological identity of nanosized materials. Nat. Nanotechnol. 7 (12), 779–786. 10.1038/nnano.2012.207 23212421

[B32] Murray P. J. Wynn T. A. (2011). Protective and pathogenic functions of macrophage subsets. Nat. Rev. Immunol. 11 (11), 723–737. 10.1038/nri3073 21997792 PMC3422549

[B33] Murugan K. Choonara Y. E. Kumar P. Bijukumar D. du Toit L. C. Pillay V. (2015). Parameters and characteristics governing cellular internalization and trans-barrier trafficking of nanostructures. Int. J. Nanomed. 10, 2191–2206. 10.2147/IJN.S75615 PMC4370919 25834433

[B34] Naz S. Beach J. Heckert B. Tummala T. Pashchenko O. Banerjee T. (2017). Cerium oxide nanoparticles: a ‘radical' approach to neurodegenerative disease treatment. Nanomed. (Lond) 12 (5), 545–553. 10.2217/nnm-2016-0399 28181459

[B35] Oró D. Yudina T. Fernández-Varo G. Casals E. Reichenbach V. Casals G. (2016). Cerium oxide nanoparticles reduce steatosis, portal hypertension and display anti-inflammatory properties in rats with liver fibrosis. J. Hepatol. 64 (3), 691–698. 10.1016/j.jhep.2015.10.020 26519601

[B36] Ould-Moussa N. Safi M. Guedeau-Boudeville M. A. Montero D. Conjeaud H. Berret J. F. (2014). In vitro toxicity of nanoceria: effect of coating and stability in biofluids. Nanotoxicology 8 (7), 799–811. 10.3109/17435390.2013.831501 23914740

[B37] Patil S. Sandberg A. Heckert E. Self W. Seal S. (2007). Protein adsorption and cellular uptake of cerium oxide nanoparticles as a function of zeta potential. Biomaterials 28 (31), 4600–4607. 10.1016/j.biomaterials.2007.07.029 17675227 PMC2259388

[B38] Pirmohamed T. Dowding J. M. Singh S. Wasserman B. Heckert E. Karakoti A. S. (2010). Nanoceria exhibit redox state-dependent catalase mimetic activity. Chem. Commun. (Camb) 46 (16), 2736–2738. 10.1039/b922024k 20369166 PMC3038687

[B39] Reed K. Cormack A. Kulkarni A. Mayton M. Sayle D. Klaessig F. (2014). Exploring the properties and applications of nanoceria: is there still plenty of room at the bottom? Environ. Sci. Nano. 1, 390–405. 10.1039/C4EN00079J

[B40] Rocca A. Moscato S. Ronca F. Nitti S. Mattoli V. Giorgi M. (2015). Pilot in vivo investigation of cerium oxide nanoparticles as a novel anti-obesity pharmaceutical formulation. Nanomedicine 11 (7), 1725–1734. 10.1016/j.nano.2015.05.001 26003299

[B41] Sanlioglu S. Williams C. M. Samavati L. Butler N. S. Wang G. McCray P. B. (2001). Lipopolysaccharide induces Rac1-dependent reactive oxygen species formation and coordinates tumor necrosis factor-alpha secretion through IKK regulation of NF-kappa B. J. Biol. Chem. 276 (32), 30188–30198. 10.1074/jbc.M102061200 11402028

[B42] Stohl W. Kaplan M. S. Gonatas N. K. (1979). A quantitative assay for experimental allergic encephalomyelitis in the rat based on permeability of spinal cords to 125I-human gamma-globulin. J. Immunol. 122 (3), 920–925. 87421

[B43] Tenzer S. Docter D. Rosfa S. Wlodarski A. Kuharev J. Rekik A. (2011). Nanoparticle size is a critical physicochemical determinant of the human blood plasma corona: a comprehensive quantitative proteomic analysis. ACS Nano. 5 (9), 7155–7167. 10.1021/nn201950e 21866933

[B44] Walkey C. D. Olsen J. B. Guo H. Emili A. Chan W. C. (2012). Nanoparticle size and surface chemistry determine serum protein adsorption and macrophage uptake. J. Am. Chem. Soc. 134 (4), 2139–2147. 10.1021/ja2084338 22191645

[B45] Xu P. T. Maidment B. W. Antonic V. Jackson I. L. Das S. Zodda A. (2016). Cerium oxide nanoparticles: a potential medical countermeasure to mitigate radiation-induced lung injury in CBA/J Mice. Radiat. Res. 185 (5), 516–526. 10.1667/RR14261.1 27135969 PMC4890072

[B47] Yokel R. A. Florence R. L. Unrine J. M. Tseng M. T. Graham U. M. Wu P. (2009). Biodistribution and oxidative stress effects of a systemically-introduced commercial ceria engineered nanomaterial. Nanotoxicology 3 (3), 234–248. 10.1080/17435390902974496

[B46] Yokel R. A. Tseng M. T. Dan M. Unrine J. M. Graham U. M. Wu P. (2013). Biodistribution and biopersistence of ceria engineered nanomaterials: size dependence. Nanomedicine 9 (3), 398–407. 10.1016/j.nano.2012.08.002 22960425

